# Serum 25-Hydroxyvitamin D Concentrations ≥40 ng/ml Are Associated with >65% Lower Cancer Risk: Pooled Analysis of Randomized Trial and Prospective Cohort Study

**DOI:** 10.1371/journal.pone.0152441

**Published:** 2016-04-06

**Authors:** Sharon L. McDonnell, Carole Baggerly, Christine B. French, Leo L. Baggerly, Cedric F. Garland, Edward D. Gorham, Joan M. Lappe, Robert P. Heaney

**Affiliations:** 1 GrassrootsHealth, Encinitas, California, United States of America; 2 Department of Family Medicine and Public Health, University of California San Diego, La Jolla, California, United States of America; 3 Department of Medicine, Creighton University, Omaha, Nebraska, United States of America; University of Alabama at Birmingham, UNITED STATES

## Abstract

**Background:**

Higher serum 25-hydroxyvitamin D [25(OH)D] concentrations have been associated with a lower risk of multiple cancer types across a range of 25(OH)D concentrations.

**Objectives:**

To investigate whether the previously reported inverse association between 25(OH)D and cancer risk could be replicated, and if a 25(OH)D response region could be identified among women aged 55 years and older across a broad range of 25(OH)D concentrations.

**Methods:**

Data from two cohorts representing different median 25(OH)D concentrations were pooled to afford a broader range of 25(OH)D concentrations than either cohort alone: the Lappe cohort (N = 1,169), a randomized clinical trial cohort (median 25(OH)D = 30 ng/ml) and the GrassrootsHealth cohort (N = 1,135), a prospective cohort (median 25(OH)D = 48 ng/ml). Cancer incidence over a multi-year period (median: 3.9 years) was compared according to 25(OH)D concentration. Kaplan-Meier plots were developed and the association between 25(OH)D and cancer risk was examined with multivariate Cox regression using multiple 25(OH)D measurements and spline functions. The study included all invasive cancers excluding skin cancer.

**Results:**

Age-adjusted cancer incidence across the combined cohort (N = 2,304) was 840 cases per 100,000 person-years (1,020 per 100,000 person-years in the Lappe cohort and 722 per 100,000 person-years in the GrassrootsHealth cohort). Incidence was lower at higher concentrations of 25(OH)D. Women with 25(OH)D concentrations ≥40 ng/ml had a 67% lower risk of cancer than women with concentrations <20 ng/ml (HR = 0.33, 95% CI = 0.12–0.90).

**Conclusions:**

25(OH)D concentrations ≥40 ng/ml were associated with substantial reduction in risk of all invasive cancers combined.

## Introduction

There were 14 million new cases of cancer worldwide in 2012 and 8.2 million cancer-related deaths [1]. Looking ahead, the annual number of new cases is projected to increase to 22 million within the next two decades [1]. In the United States, it is estimated that over 1.68 million new cases of cancer will be diagnosed in 2016 and almost 600,000 deaths due to cancer will occur [1]. A total of $125 billion was spent on cancer care in the United States in 2010, and is expected to grow to over $150 billion in 2020 [1]. A focus on primary prevention is imperative to slow or reverse these upward trends in cancer incidence, treatment burden, mortality, and associated costs.

Thirty-five years ago, Garland and Garland first proposed a link between cancer and vitamin D from observations of higher colon cancer mortality in higher latitudes and areas with less solar radiation [2]. Since then, multiple epidemiologic studies have found an inverse association between serum 25-hydroxyvitamin D [25(OH)D] concentration and the risk of many types of cancer including breast [3–10], colorectal [11–12], and prostate [13]. In a randomized controlled trial by Lappe et al. [14], it was found that women assigned to a vitamin D and calcium treatment group had a 60% reduction in incidence of all non-skin cancers compared to women in the placebo group (RR = 0.40, 95% CI: 0.20–0.82, *P* = 0.01). For women free of cancer at one year into the trial, the reduction in incidence was 77% (RR = 0.23, 95% CI: 0.09–0.60, *P*<0.005).

The objective of this analysis was to quantify more precisely the association between 25(OH)D concentration and risk of non-skin cancer among women aged 55 years and older, and to do so across a broader range of 25(OH)D concentrations than had been previously analyzed. The present study used data from two cohorts representing different median 25(OH)D concentrations. The first was a cohort from a randomized controlled clinical trial performed by Lappe et al. [14,15] in Nebraska (N = 1,169), with a median 25(OH)D of 30 ng/ml (Interquartile Range (IQR): 25–37). The Lappe et al. cohort (hereafter termed Lappe cohort) provided a majority of the data for the lower 25(OH)D concentrations. The second was a cohort from a prospective cohort study consisting of volunteer participants residing in 52 countries worldwide (90% in the United States or Canada) recruited by a non-profit public health research organization, GrassrootsHealth of San Diego, California (N = 1,135), with a median 25(OH)D of 48 ng/ml (IQR: 39–61). The GrassrootsHealth cohort provided a majority of the data for the higher 25(OH)D concentrations. Because of the larger sample size, this pooled cohort has improved statistical power. It also provided a broader range of 25(OH)D concentrations than either cohort alone. This novel approach allowed for analysis of results across a wide range of 25(OH)D concentrations. This would otherwise not have been possible due to the paucity of data in the higher concentrations of 25(OH)D in the Lappe and other similar treatment cohorts. Serum 25(OH)D concentration was selected as the independent variable rather than treatment assignment or reported intake because it is a better indicator of vitamin D status, capturing the effect of multiple input sources and making provision for inter-individual variability in dose response [16].

## Materials and Methods

### Lappe Cohort

The Lappe cohort participated in a four year, double-blind, placebo-controlled trial of vitamin D and calcium supplementation. A detailed description of the participants and study design can be found elsewhere [14,15]. Briefly, participants were recruited via random digit dialing within a 9-county area in Eastern Nebraska as a population-based sample. Inclusion criteria included women aged 55 years and older without known cancer at enrollment or within 10 years prior. All participants were non-Hispanic white. Participants who were lost to follow-up before their second visit were excluded because of lack of prospective data for this analysis. All participants provided written informed consent and this research study was approved by the Creighton University Institutional Review Board (Omaha, NE).

Participants were randomly assigned to one of three groups: calcium (either 1400 mg/day of calcium citrate or 1500 mg/day of calcium carbonate, plus vitamin D placebo), calcium plus vitamin D (calcium as mentioned previously plus 1000 IU/day of vitamin D3), or control (calcium and vitamin D placebos). The calcium group and the calcium plus vitamin D group each comprised 40% of the total cohort, with 20% serving as the control group. Health status and supplement intake according to bottle weight were assessed at 6-month interval visits. When a diagnosis of cancer was reported, medical records were examined to confirm diagnosis and ascertain diagnosis date. Serum 25(OH)D concentrations were measured at baseline and annually thereafter using radioimmunoassay after extraction with the use of the IDS Radioimmunoassay kit (Foundation Hills, AZ) at the Creighton University Osteoporosis Research Center Laboratory (Omaha, NE). The laboratory participates in the Vitamin D Quality Assessment Scheme (DEQAS), whose objective is to ensure the analytical reliability of 25(OH)D assays, with findings on test samples collected during the course of the study regularly close to the international mean.

### GrassrootsHealth Cohort

GrassrootsHealth, a non-profit public health research organization, has been running a large prospective population-based study allowing voluntary participants to reach and sustain a serum 25(OH)D concentration of their choice, and tracking self-reported health status measures through a questionnaire. Participants were individuals who responded to an invitation to attendees at a GrassrootsHealth seminar in 2008 and others recruited via internet. There were no exclusion criteria for enrollment. Participation required submission of a home blood spot 25(OH)D test kit and completion of an online health questionnaire. All participants provided informed consent and this research study was approved by the Western Institutional Review Board (Olympia, WA).

This current analysis included all non-Hispanic white female participants aged 55 years and older without known cancer at enrollment or within 10 years prior who completed at least two health assessments and 25(OH)D measurements. These inclusion criteria were chosen to match GrassrootsHealth members to features of the Lappe cohort. Between January 2009 and December 2014, participants completed health questionnaires and home blood spot test kits at approximately 6 month intervals. Cancer diagnosis dates and types were reported as well as average daily calcium supplement intake, smoking status, and height and weight for calculation of body mass index (BMI). Serum 25(OH)D concentrations were determined by blood spot test kits analyzed using liquid chromatography-mass spectroscopy (LC-MS/MS) by ZRT Laboratory (Beaverton, OR) or Purity Laboratory (Lake Oswego, OR). Both the ZRT and Purity assays had been validated against the LC-MS/MS consensus group reporting to DEQAS, with *R*^2^ values of 0.998 and 0.994 respectively. LC-MS/MS has been validated against the radioimmunoassay method, with an *R*^2^ value of 0.91 and with a slope not different from 1.0 [17].

Overall, this analysis included 1,169 women from the Lappe cohort (median follow-up time, 4.0 years) and 1,135 women from the GrassrootsHealth cohort (median follow-up time, 1.2 years) (pooled cohort N = 2,304; median follow-up time, 3.9 years). The total person-time was 4,239 person-years in the Lappe cohort and 2,175 person-years in the GrassrootsHealth cohort (6,414 person-years in the pooled cohort). The most common type of cancer diagnosed during the study was breast cancer (43% of all cancers in the pooled cohort).

### Statistical Methods

Demographic characteristics for the Lappe and GrassrootsHealth cohorts were summarized and compared using Mann-Whitney tests for age, BMI, and baseline 25(OH)D, and the chi-square test was used to compare smoking status. Mean 25(OH)D concentration was calculated for each participant by taking the mean of all 25(OH)D measurements during the observation period (for cases, only measurements before the date of diagnosis were used). The outcome of interest was the diagnosis of any non-skin cancer during the period of observation. A previous analysis of the Lappe cohort [14] included 2 cases of melanoma but these were considered skin cancer and not identified as cases for this analysis. Age-adjusted incidence rates (standardized to the 2010 US population) of non-skin cancer (hereafter termed cancer) were calculated for the Lappe and GrassrootsHealth cohorts.

Cancer incidence rates and 95% confidence intervals were calculated for successive 20 ng/ml groups of 25(OH)D concentration, using a moving average method [18–20] to assess incidence trends across the range of 25(OH)D for both baseline 25(OH)D and mean 25(OH)D (mean of all measurements for a given individual during the observation period as a single measure of overall vitamin D status during the study). Using the curve fitting routine of SigmaPlot 12.3 (Systat Software Inc., San Jose, CA, USA), the relationship of serum 25(OH)D concentration to cancer incidence was fitted to an exponential equation (Y = A + B(e^(-X/C)^), where Y = cancer incidence rate, X = serum 25(OH)D).

To estimate cancer-free survival over time and account for varying lengths of follow-up, Kaplan-Meier cancer-free survival curves were developed (allowing for participants switching groups). The proportion of cancer-free participants for 25(OH)D concentrations of <20 ng/ml, 20–39 ng/ml, and ≥40 ng/ml were computed.

Multivariate Cox regression was used to determine the association between serum 25(OH)D and the risk of developing cancer, adjusting for age, BMI, smoking status, and calcium supplement intake. Hazard ratio (HR) estimates and 95% confidence intervals (CIs) were calculated for 25(OH)D concentration as a categorical variable divided into three a priori categories: <20 ng/ml, 20–39 ng/ml, ≥40 ng/ml (the 20 ng/ml cut point is from the Institute of Medicine (IOM) recommendation for bone health [21] and the 40 ng/ml cut point is from literature recommending at least this concentration for cancer prevention [22–26]). Calcium supplement intake was also assessed as a categorical variable (<1000 mg/day vs. ≥1000 mg/day) based on the IOM recommendation for bone health [21]. Since 25(OH)D concentration and calcium supplement intake changed over the course of the study for most participants, multiple values for each participant were entered in the model as time varying, with their mean values for the prior year. Age and BMI were entered as baseline continuous variables and smoking status was entered as a categorical variable (yes/no) for “current smoker” at baseline. The proportional hazards assumption was tested by assessing the interaction of each variable with log of time. Effect modification was tested by including pair-wise product interaction terms. Modeling was confined to participants with valid values for all of the involved variables.

The association between 25(OH)D concentration and cancer risk was assumed to be non-linear, based on the typical nutrient physiological response which consists of a response region where improved nutrient status produces significantly lower risk, and an upper region where less benefit is observed with increasing status [27]. A multivariate Cox regression model with restricted cubic splines with 3 knots in default locations was used to identify the nature of the non-linear association between 25(OH)D and cancer risk. Analyses were performed using the R software (www.r-project.org).

## Results

The median baseline serum 25(OH)D concentration in the Lappe cohort was 28 ng/ml (IQR: 23–34) and in the GrassrootsHealth cohort was 43 ng/ml (IQR: 34–58) (*P*<0.0001). The baseline characteristics of the Lappe and GrassrootsHealth cohorts are shown in Table 1. The Lappe cohort had a higher median age and BMI and a higher proportion of participants who were current smokers.

**Table 1:** Demographic characteristics of the pooled, Lappe, and GrassrootsHealth cohorts.

	Pooled Cohort (N = 2304)	Lappe Cohort (N = 1169)	GrassrootsHealth Cohort (N = 1135)	P-value^a^ (comparing Lappe vs. GrassrootsHealth)
**Age (years)**: Median (IQR)	64 (60–69)	66 (60–71)	62 (59–66)	<0.0001
**BMI**: Median (IQR)	26 (23–31)	28 (25–32)	24 (21–28)	<0.0001
**Smoking Status**: N (%)				<0.0001
Current	138 (6%)	107 (9%)	31 (3%)	
Never/Former	2165 (94%)	1062 (91%)	1103 (97%)	
**Baseline 25(OH)D (ng/ml)**: Median (IQR)	34 (26–44)	28 (23–34)	43 (34–58)	<0.0001
**Mean 25(OH)D (ng/ml)**^b^: Median (IQR)	37 (29–49)	30 (25–37)	48 (39–61)	<0.0001

^a^Statistical comparison of characteristics between Lappe and GrassrootsHealth cohorts. Age, BMI, baseline 25(OH)D, and mean 25(OH)D were compared using Mann-Whitney tests. Smoking status was compared using chi-square test.

^b^Mean 25(OH)D concentration was computed for each participant as the mean of all 25(OH)D measurements during the observation period as a single measure of overall 25(OH)D concentration status during the study.

Mean 25(OH)D concentration was computed for each participant as the mean of all 25(OH)D measurements during the observation period as a single measure of overall 25(OH)D concentration status during the study. The median of the within-subject mean 25(OH)D values, calculated for each cohort, was 30 ng/ml (IQR: 25–37) in the Lappe cohort and 48 ng/ml (IQR: 39–61) in the GrassrootsHealth cohort (*P*<0.0001). Corresponding values for the pooled cohort were 37 ng/ml (IQR: 29–49). 55% of women in the Lappe cohort and 17% in the GrassrootsHealth cohort had a mean calcium supplement intake of ≥1000 mg/day (*P*<0.0001) (36% in the pooled cohort). Those in the Lappe cohort with a mean calcium supplement intake of <1000 mg/day were either assigned to the control group or took <70% of assigned calcium doses.

Fifty-eight women in the pooled cohort were diagnosed with cancer during the observation periods (48 from the Lappe cohort and 10 from the GrassrootsHealth cohort). The age-adjusted incidence rate of cancer was 840 cases per 100,000 person-years in the pooled cohort, 1020 cases per 100,000 person-years in the Lappe cohort and 722 cases per 100,000 person-years in the GrassrootsHealth cohort. Cancer types for each cohort are shown in S1 Table.

**Fig 1:**
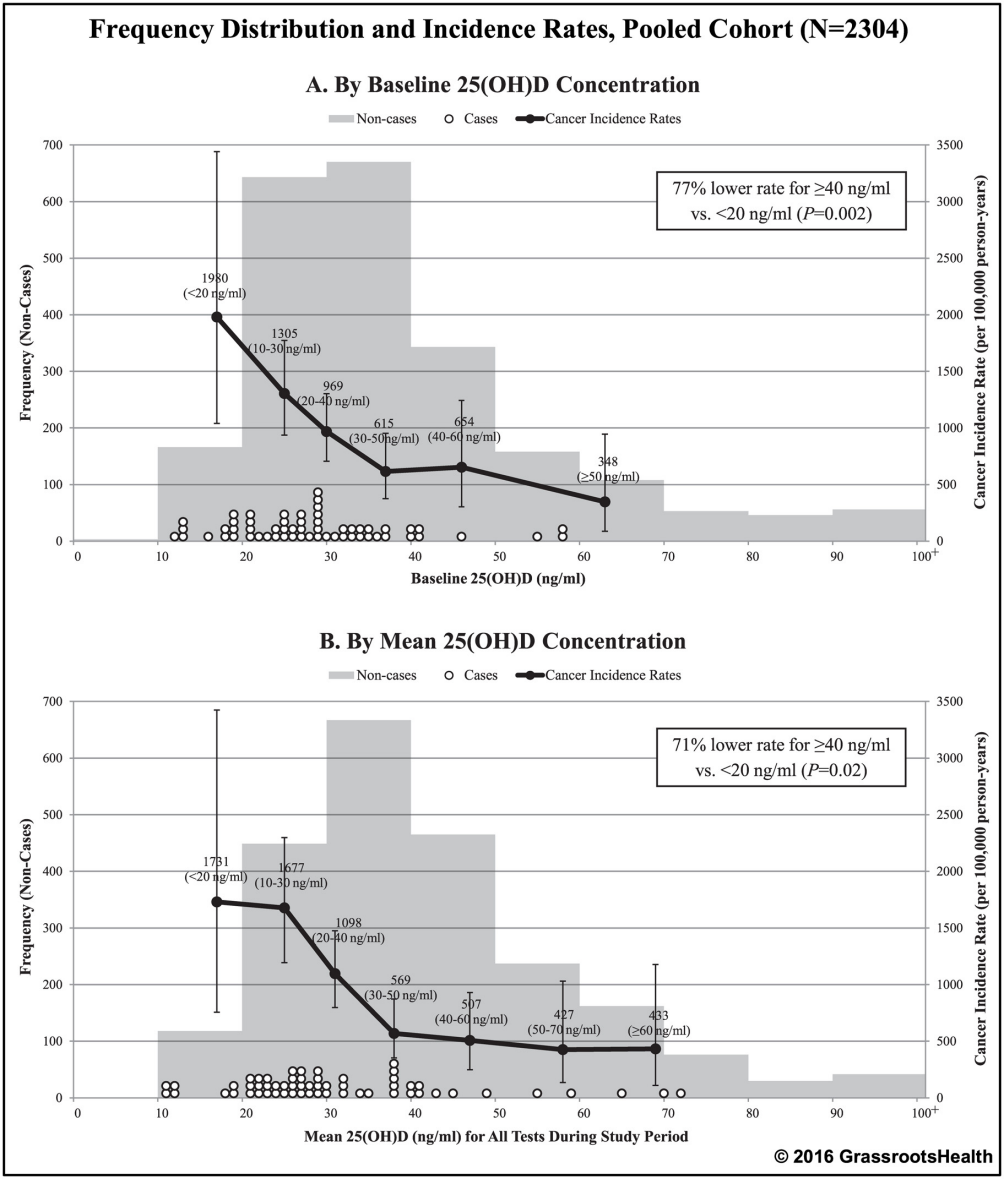
Frequency distribution and cancer incidence rates by 25(OH)D concentration, pooled cohort (N = 2304). The bars represent the number of non-cases by groupings of 10 ng/ml, white dots represent the 25(OH)D concentration for each cancer case, black dots represent cancer incidence rates per 100,000 person-years for indicated 25(OH)D groupings (plotted at the median 25(OH)D value for each grouping: baseline 25(OH)D groups at 17, 25, 30, 37, 46, and 63 ng/ml; mean 25(OH)D groups at 17, 25, 31, 38, 47, 58, and 69 ng/ml). Vertical error bars indicate the 95% confidence intervals.

**Fig 2:**
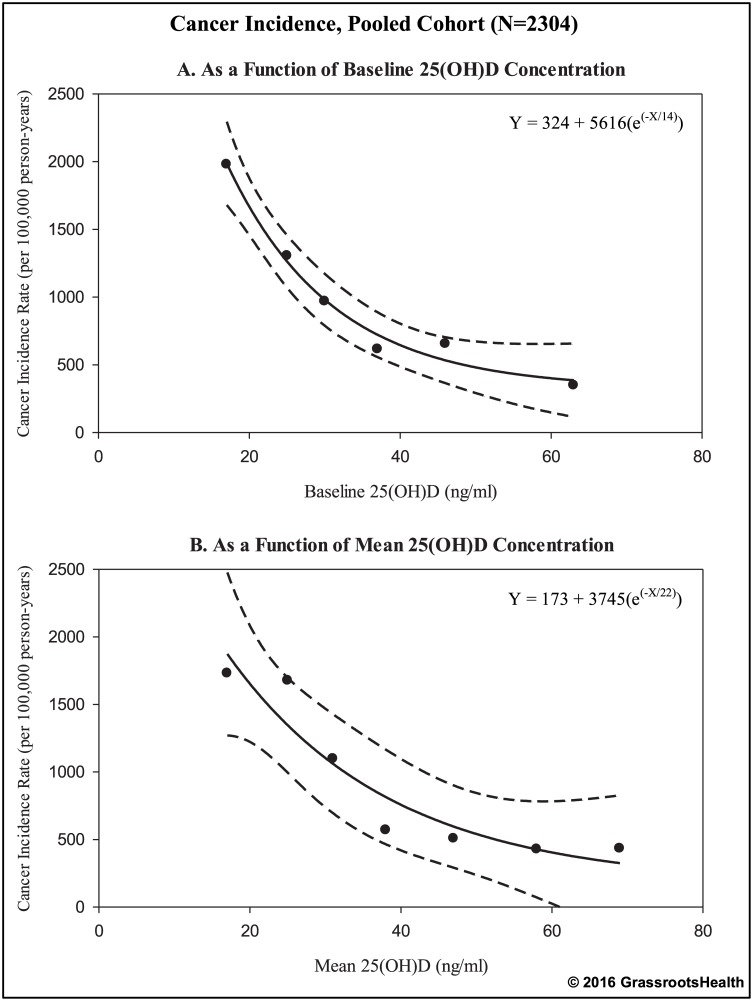
Cancer incidence rates by 25(OH)D concentration with fitted curves, pooled cohort (N = 2304). Black dots represent cancer incidence rates per 100,000 person-years for indicated 25(OH)D groupings (rates are displayed at the median value for each grouping: baseline 25(OH)D groups at 17, 25, 30, 37, 46, and 63 ng/ml; mean 25(OH)D groups at 17, 25, 31, 38, 47, 58, 69 ng/ml), as seen in Fig 1. Solid black lines represent the best fit line to the exponential equation: Y = A + B(e^(-X/C)^), where Y = cancer incidence rate, X = serum 25(OH)D (dashed lines represent the 95% confidence intervals).

To assess cancer incidence trends across the range of 25(OH)D in the pooled cohort, incidence rates were calculated according to categories of 25(OH)D for both baseline 25(OH)D and mean 25(OH)D (mean of all measurements during the observation period as a measure of overall 25(OH)D concentration during the study) (Fig 1). For both baseline and mean 25(OH)D, when 25(OH)D was higher, incidence rates were lower. Specifically, there was a 77% lower incidence rate of cancer for ≥40 ng/ml vs. <20 ng/ml for baseline 25(OH)D (Rate Ratio = 0.23, 95% CI: 0.09–0.59, *P* = 0.002) and a 71% lower incidence rate for mean 25(OH)D (Rate Ratio = 0.29, 95% CI: 0.11–0.77, *P* = 0.02). Fig 2 shows plots of cancer incidence rates by baseline and mean serum 25(OH)D with fitted exponential curves. Rates were lower in higher 25(OH)D categories (≥40 ng/ml). There was continued gradual decline with higher concentrations.

**Fig 3:**
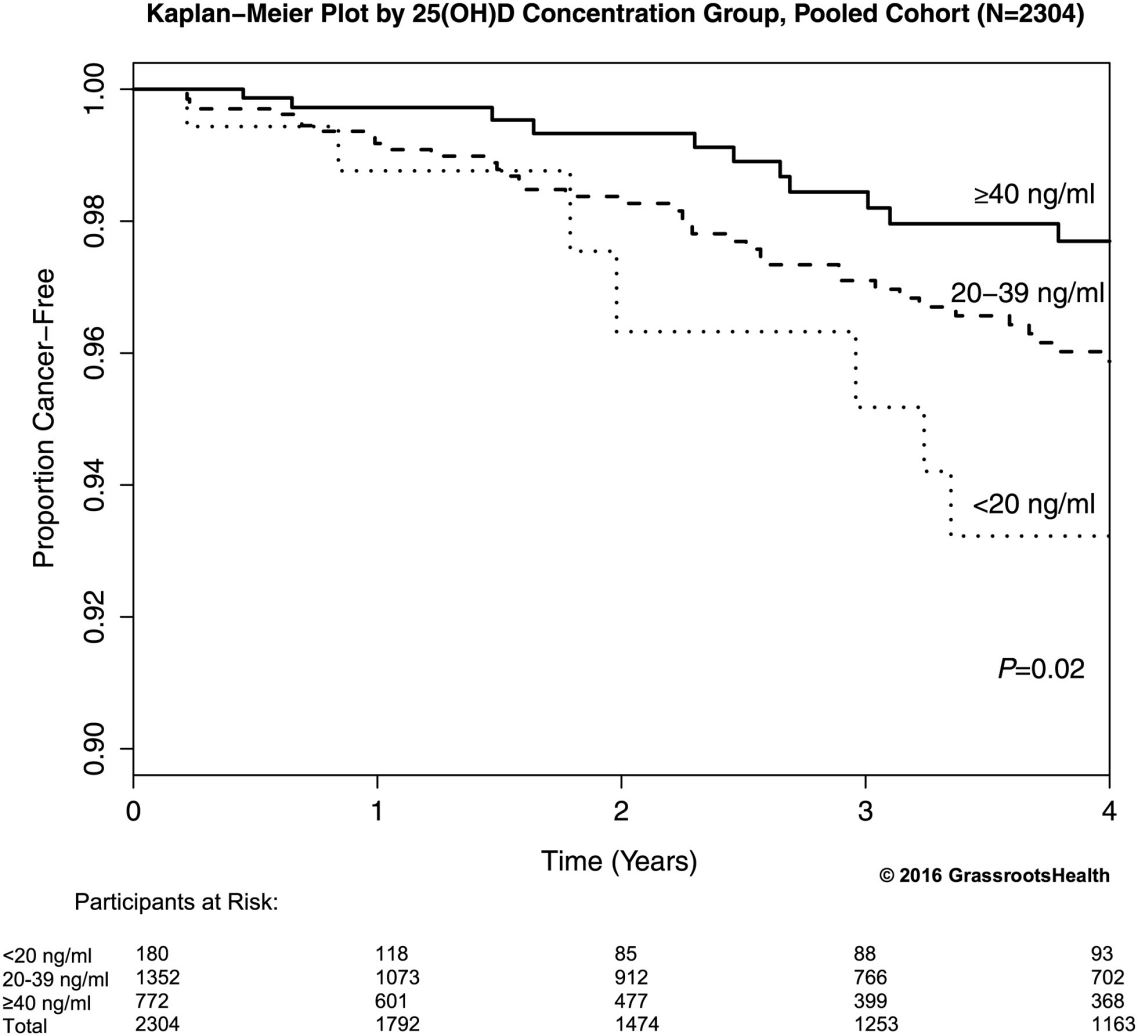
Kaplan-Meier plot comparing the proportion of cancer-free participants by 25(OH)D concentration (allowing for participants switching groups), pooled cohort (N = 2304). Four-year cumulative cancer-free proportion was 98% among participants with 25(OH)D concentrations ≥40 ng/ml compared to 93% for those with 25(OH)D concentrations <20 ng/ml (proportion with cancer was 71% lower for ≥40 ng/ml vs. <20 ng/ml, *P* = 0.02).

To estimate cancer-free survival over time and account for varying lengths of follow-up, Kaplan-Meier curves comparing the proportion of cancer-free participants for <20 ng/ml, 20–39 ng/ml, and ≥40 ng/ml (allowing for participants switching groups) were computed for the pooled cohort (Fig 3). These curves were significantly different, with the highest proportion cancer-free at 4 years in the ≥40 ng/ml group (98%) and the lowest proportion cancer-free in the <20 ng/ml group (93%) (proportion with cancer was 71% lower for ≥40 ng/ml vs. <20 ng/ml, *P* = 0.02). The ≥40 ng/ml group diverged early from the other groups and the 20–39 ng/ml diverged from the <20 ng/ml group at approximately 2 years.

Multivariate Cox regression was used to quantify the association between 25(OH)D and cancer risk, adjusting for other risk factors (allowing multiple 25(OH)D values per participant to accommodate changes over the course of the study). Women in the pooled cohort with 25(OH)D concentrations ≥40 ng/ml had a 67% lower risk of cancer compared to women with concentrations <20 ng/ml, adjusting for age, BMI, smoking status, and calcium supplement intake (HR = 0.33, 95% CI: 0.12–0.90, *P* = 0.03) (Table 2). Those with concentrations of 20–39 ng/ml had 43% lower risk of cancer compared to those with concentrations <20 ng/ml (HR = 0.57, 95% CI: 0.25–1.30, *P* = 0.18). Women taking ≥1000 mg/day of calcium supplements had a 19% lower risk of cancer, but this association was not significant (HR = 0.81, 95% CI: 0.47–1.40, *P* = 0.45); also, the association of 25(OH)D with cancer risk did not vary based on the level of calcium supplement intake. Age, BMI, and smoking status were also not significant predictors of cancer risk.

**Table 2:** Association between serum 25(OH)D and risk of cancer, pooled cohort (N = 2304).

Serum 25(OH)D (ng/ml)	Unadjusted Hazard Ratio (95% CI)	p-value	Adjusted^a^ Hazard Ratio (95% CI)	p-value
<20	Reference		Reference	
20–39	0.61 (0.27,1.36)	0.22	0.57 (0.25,1.30)	0.18
≥40	**0.33 (0.13,0.85)**	**0.02**	**0.33 (0.12,0.90)**	**0.03**

Bold values signify significant hazard ratios.

^a^Adjusted for age, BMI, smoking status, and calcium supplement intake.

**Fig 4:**
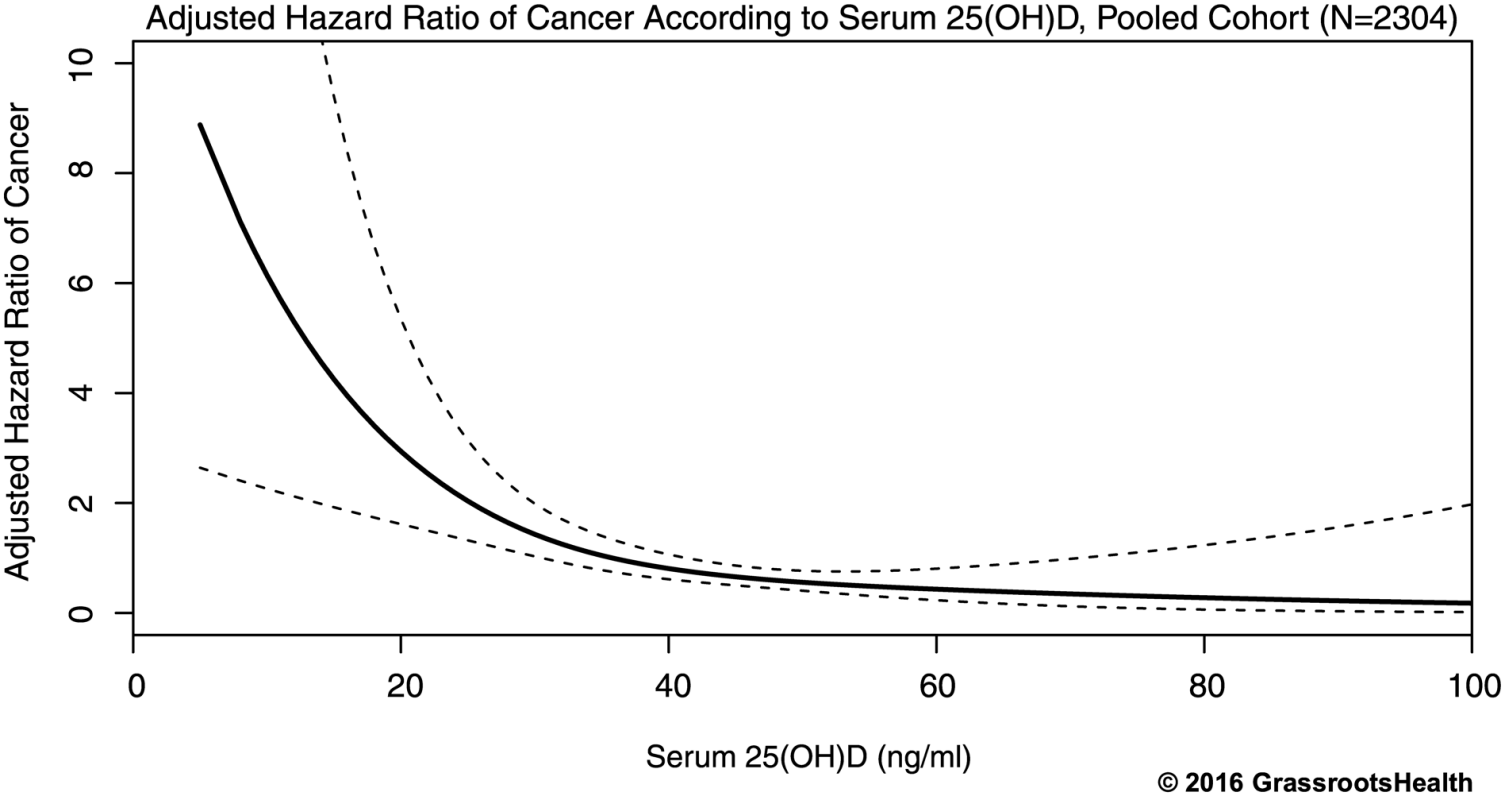
Association between serum 25(OH)D and risk of cancer adjusted for age, BMI, smoking status, and calcium supplement intake in the range of ≤100 ng/ml, pooled cohort (N = 2304). Solid black line represents the estimated hazard ratio for the Cox regression model with restricted cubic splines with 3 knots and dashed lines represent the 95% confidence interval of the estimate. The reduction in risk from 20 ng/ml to 40 ng/ml was approximately 70%.

Multivariate Cox regression with spline terms was used to identify the nature of the non-linear association between 25(OH)D and cancer risk. Spline regression revealed a sharp decrease in the risk of cancer with increased 25(OH)D concentration in the lower range of 25(OH)D and a gradual decline as serum concentrations neared 40 ng/ml and above, with no evidence of increased risk in the upper 25(OH)D concentrations (Fig 4). The reduction in risk from 20 ng/ml to 40 ng/ml was approximately 70%.

Findings were similar when we limited the analysis to just the Lappe cohort (S1–S3 Figs and S2 Table) and when we excluded non-US residents in the GrassrootsHealth cohort. Additionally, we conducted multivariate Cox regression analysis using tertiles of 25(OH)D concentration and the results were also similar. Specifically, women with 25(OH)D concentrations in the highest tertile (≥40 ng/ml) had a 65% lower risk of cancer compared to women with concentrations in the lowest tertile (<28 ng/ml) (HR = 0.35, 95% CI: 0.17–0.75, *P* = 0.007). In the Lappe cohort, women in the highest tertile (≥32 ng/ml) had a 61% lower risk of cancer compared to women with concentrations in the lowest tertile (<25 ng/ml) (HR = 0.39, 95% CI: 0.18–0.84, *P* = 0.02).

## Discussion

We found a clear association between 25(OH)D serum concentration and cancer risk, according to multiple types of analyses. These results suggest the importance of vitamin D for the prevention of cancer. Women with 25(OH)D concentrations ≥40 ng/ml had a significantly lower risk of cancer (~70%) compared to women with concentrations <20 ng/ml. When looking at the risk of cancer across the 25(OH)D continuum available in the pooled cohort, we observed the greatest decrease in risk occurring between ~10–40 ng/ml, with additional benefit also observed at ≥40 ng/ml. We would have preferred to have randomized clinical trial data in the higher 25(OH)D ranges, but since these do not currently exist, using the GrassrootsHealth prospective cohort data allowed us to observe results across a wider range of 25(OH)D concentrations than using the Lappe cohort alone.

Other studies have found a similar reduction in risk for individual cancers [3–13]. Lowe et al. demonstrated in a hospital-based case control study that women with serum concentrations of >60 ng/ml had an 83% reduction in breast cancer risk compared to women with concentrations <20 ng/ml (*P*<0.001) [3]. A population-based case control study found a 63% lower risk of breast cancer for women with 25(OH)D concentrations ≥30 ng/ml compared to women with concentrations <20 ng/ml (OR = 0.37, 95% CI: 0.27–0.51), with a 71% lower risk among post-menopausal women (OR = 0.29, 95% CI: 0.19–0.45) [6]. A recent nested case-control study found a 55% lower risk of colorectal cancer in women with 25(OH)D concentrations ≥29 ng/ml compared to women with concentrations <18 ng/ml (OR = 0.45, 95% CI: 0.25–0.81) [12].

Two previous studies of the Women’s Health Initiative (WHI) randomized trial did not find an association between vitamin D treatment group and colorectal or breast cancer risk [28,29]. That trial used vitamin D doses of 400 IU/day, an intake amount that is unlikely to raise basal concentrations to a sufficient status, and experienced poor compliance (approximately 50%). When the WHI data were analyzed by baseline 25(OH)D concentration, higher 25(OH)D was associated with lower cancer risk (60% lower risk of colorectal cancer for ≥23 ng/ml vs. <12 [28] and 78% lower risk of colorectal cancer for ≥26 ng/ml vs. <13 ng/ml [29]). Also, studies of other cancers from this trial found significant inverse relationships between vitamin D status and lung and pancreatic cancer risk [30,31].

Future studies should base their study design on nutrient physiology, taking into account the sigmoidal nature of nutrient response by assessing participants whose basal status was deficient (<20 ng/ml) and using a vitamin D dose sufficient to raise 25(OH)D concentrations to above the response range (~40 ng/ml as seen in Fig 4) [27]. Comparing 25(OH)D concentration groups outside of this response range or treating the relationship as linear, spread across the entire 25(OH)D range instead of a sigmoidal response would dilute the effect. Further, comparing 25(OH)D concentration groups over too narrow a region of the response range may also yield non-significant results. Note in Fig 4 the smallness in the reduction in risk as 25(OH)D exposure increases from 25 to 35 ng/ml. To the extent that Fig 4 adequately represents the association of risk and vitamin D status, this small effect size in the mid exposure range may explain why many treatment studies, particularly those that did not raise mean 25(OH)D concentration into the range above 40 ng/ml, failed to detect an association of serum 25(OH)D with cancer. This illustrates the importance of dosing in a manner calculated to span an appreciable portion of the sigmoid response curve [27].

Of interest, several newly-identified, naturally occurring vitamin D3 metabolites have been detected in human epidermis and serum that are products of CYP11A1-mediated metabolism of vitamin D [32]. These vitamin D metabolites exert anti-proliferative, pro-differentiation, and anti-inflammatory effects that are similar to, or greater than, the effects of 1,25(OH)_2_D3 [33,34]. Also, the major vitamin D metabolite from this pathway, 20(OH)D3, occurs in much higher concentrations in human serum than 1,25(OH)_2_D3 (~60 times) [34]. This new research shows that there are many active metabolites of vitamin D that may be contributing to the influence of solar UVB and serum 25(OH)D3. These newly-identified metabolites should be considered for inclusion in future studies of cancer prevention since their spectrum of action may extend beyond that of 25(OH)D3 and 1,25(OH)_2_D3.

Calcium supplement intake was not a significant independent predictor of cancer risk in this analysis. This finding is similar to the results of the previous analysis based on assigned treatment group by Lappe et al., that found that women assigned to the calcium treatment group did not have a significant reduction in cancer risk compared to women in the control group (RR = 0.53, 95% CI: 0.27–1.03), including among the subgroup of women who were free of cancer at one year (RR = 0.59, 95% CI: 0.29–1.21) [14]. Since information on dietary calcium intake was unavailable, the present analysis was limited to the assessment of supplemental calcium intake. It is possible that dietary calcium intake (or total calcium intake) may play a role in cancer risk.

The median follow-up time for the Lappe cohort was 4.0 years, compared to 1.2 years for the GrassrootsHealth cohort. All rates were calculated using person-time denominators, so the difference in length of follow-up did not affect the rates. Also, Kaplan-Meier plots and Cox regression were used because these analyses account for varying lengths of follow-up. Still, there could be a difference between the two cohorts if a certain amount of time must pass for the serum 25(OH)D to exert its influence. For example, in the original analysis of the Lappe cohort [14], the effect of vitamin D on risk of all invasive cancers was greater if one year was allowed to pass before starting the counting of cases. If we assume that participants of the GrassrootsHealth cohort had started their self-intervention less than a year before follow-up began, the true association might have been stronger if a longer time interval was available. The impact of vitamin D on risk of cancer could be greater than the hazard ratio we report here (HR = 0.33, 95% CI = 0.12–0.90) (Table 2). The GrassrootsHealth cohort is still under active surveillance, and a future article will report on the effect of longer follow-up.

There were three times as many cases of breast cancer in the Lappe cohort than in the GrassrootsHealth cohort; however, median follow-up time for the Lappe cohort was 3.3 times longer than for the GrassrootsHealth cohort. Also, the GrassrootsHealth cohort had higher 25(OH)D concentrations than the Lappe cohort, which has been associated with lower risk of breast cancer [3–10]. It is of some interest that there were seven cases of lung cancer in the Lappe cohort compared to zero cases in the GrassrootsHealth cohort. Although the difference was only statistically significant at *P* = 0.11, and could be explained by alternative explanations, it is consistent with the existence of an inverse relationship between vitamin D status and risk of lung cancer [30,35]. Some of the differences may also have been due to the median age of the Lappe cohort being 4 years older than that of the GrassrootsHealth cohort, the BMI of the Lappe cohort being 28 compared to 24 in the GrassrootsHealth cohort, and the proportion of current smokers in the Lappe cohort being 9% compared to 3% in the GrassrootsHealth cohort. However, this analysis took into account these cohort differences when assessing the association between 25(OH)D and cancer risk by adjusting for these factors in multivariate Cox regression analyses (Table 2). Further follow-up of the GrassrootsHealth cohort may result in larger numbers of cases for particular cancer types, allowing analysis for individual cancer types.

The strengths of this analysis include using serum 25(OH)D concentration, which is the physiological measure of vitamin D status accounting for all input sources including cutaneous, supplementation, and food. Using serum 25(OH)D concentration also overcomes the inherent bias of treatment compliance and inter-individual variability in dose response which are features of analyses based on assignment to treatment groups. Also, entering 25(OH)D concentration and calcium supplement intake as separate covariates allowed us to assess the independent roles of vitamin D and calcium on cancer risk. This analysis also used multiple analysis techniques, including comparing cancer incidence across 25(OH)D groups, computing Kapan-Meier plots, and conducting adjusted Cox regression analyses with multiple 25(OH)D measurements per participant to account for changes in vitamin D status, all of which found a >65% lower risk or rate of cancer for those with 25(OH)D concentrations ≥40 ng/ml compared to those with concentrations <20 ng/ml. Additionally, this analysis included individuals with a wider range of serum 25(OH)D concentrations than other studies and identified the response region of cancer risk related to 25(OH)D.

Limitations of this study include the use of self-report data for certain variables where recall bias may have occurred. This analysis also was not able to control for some covariates related to cancer risk such as family history of cancer, diet, physical activity, or alcohol use. Differences in methods and demographics between the Lappe and GrassrootsHealth cohorts may have affected pooled analyses, which is why results for the Lappe cohort were also presented separately. The small proportion of current smokers in the study population did not allow for an adequate assessment of the association between smoking status and cancer risk, a known risk factor for cancer. However, this provided a unique opportunity to assess other risk factors without the potentially overpowering influence of smoking. An additional limitation is that we did not have sufficient power to assess associations for individual cancer types. While this analysis did show a significant inverse association of 25(OH)D concentration and risk of cancer for non-Hispanic white women aged 55 years and older, results may not be generalizable to other ethnicities, age groups, or to males.

The findings from this analysis support the inverse association between 25(OH)D and risk of cancer and highlight the importance for cancer prevention of achieving a concentration substantially above 20 ng/ml, the concentration recommended by the IOM for bone health [21]. Increasing 25(OH)D concentrations to a minimum of 40 ng/ml could substantially reduce cancer incidence and associated mortality in the population based on these findings as well as other studies [22–26]. Primary prevention of cancer, rather than solely expanding early detection or improving treatment, will be essential for reversing the current upward trend of cancer incidence worldwide; this analysis suggests that improving vitamin D status is a key prevention tool.

## Supporting Information

S1 Fig Frequency distribution and cancer incidence rates by 25(OH)D concentration, Lappe cohort (N = 1169). The bars represent the number of non-cases by groupings of 10 ng/ml, white dots represent the 25(OH)D concentration for each cancer case, black dots represent cancer incidence rates per 100,000 person-years for indicated 25(OH)D groupings (plotted at the median 25(OH)D value for each grouping: baseline 25(OH)D groups at 17, 24, 29, 35, and 43 ng/ml; mean 25(OH)D groups at 17, 25, 30, 36, and 43 ng/ml). Vertical error bars indicate the 95% confidence intervals.
(EPS)

S2 Fig Kaplan-Meier plot comparing the proportion of cancer-free participants by 25(OH)D concentration (allowing for participants switching groups), Lappe cohort (N = 1169). Four-year cumulative cancer-free proportion was 97% among participants with 25(OH)D concentrations ≥40 ng/ml compared to 93% for those with 25(OH)D concentrations <20 ng/ml (proportion with cancer was 57% lower for ≥40 ng/ml vs. <20 ng/ml, *P* = 0.16).
(EPS)

S3 Fig Association between serum 25(OH)D and risk of cancer adjusted for age, BMI, smoking status, and calcium supplement intake in the range of ≤50 ng/ml, Lappe cohort (N = 1169). Solid black line represents the estimated hazard ratio for the Cox regression model with restricted cubic splines with 3 knots and dashed lines represent the 95% confidence interval of the estimate. The reduction in risk from 20 ng/ml to 40 ng/ml was approximately 80%.
(EPS)

S1 Table Cancer types for pooled, Lappe, and GrassrootsHealth cohorts. (DOCX)

S2 Table Association between serum 25(OH)D and risk of cancer, Lappe cohort (N = 1169). (DOCX)
