# Correction: Serum 25-Hydroxyvitamin D Concentrations ≥40 ng/ml Are Associated with >65% Lower Cancer Risk: Pooled Analysis of Randomized Trial and Prospective Cohort Study

**DOI:** 10.1371/journal.pone.0201078

**Published:** 2018-07-16

**Authors:** Sharon L. McDonnell, Carole Baggerly, Christine B. French, Leo L. Baggerly, Cedric F. Garland, Edward D. Gorham, Joan M. Lappe, Robert P. Heaney

The Data Availability statement for this paper is incorrect. The correct statement is: The data are available through the Zenodo repository and can be accessed at https://doi.org/10.5281/zenodo.1299364.
