# Supplementary material for: Serum 25-Hydroxyvitamin D Concentrations ≥40 ng/ml Are Associated with >65% Lower Cancer Risk: Pooled Analysis of Randomized Trial and Prospective Cohort Study
Source: PLoS One. 2016 Apr 6;11(4):e0152441. doi: 10.1371/journal.pone.0152441 (PMC4822815; doi:10.1371/journal.pone.0152441)
Supplement: S1 Table — (DOCX) [file pone.0152441.s004.docx]

**S1 Table. Cancer types for pooled, Lappe, and GrassrootsHealth cohorts**

|  | **Pooled Cohort (N=2304)** | **Lappe Cohort (N=1169)** | **GrassrootsHealth Cohort (N=1135)** |
| --- | --- | --- | --- |
| **Breast** | 25 | 19 | 6 |
| **Colon** | 6 | 3 | 3 |
| **Laryngeal** | 1 | 1 | 0 |
| **Leukemia** | 3 | 3 | 0 |
| **Lung** | 7 | 7 | 0 |
| **Lymphoma** | 4 | 4 | 0 |
| **Multiple Myeloma** | 2 | 2 | 0 |
| **Ovarian** | 1 | 1 | 0 |
| **Pancreatic** | 1 | 1 | 0 |
| **Renal** | 2 | 2 | 0 |
| **Thyroid** | 2 | 1 | 1 |
| **Urethral** | 1 | 1 | 0 |
| **Uterine** | 3 | 3 | 0 |
| **Total** | 58 | 48 | 10 |
