# Supplementary material for: Serum 25-Hydroxyvitamin D Concentrations ≥40 ng/ml Are Associated with >65% Lower Cancer Risk: Pooled Analysis of Randomized Trial and Prospective Cohort Study
Source: PLoS One. 2016 Apr 6;11(4):e0152441. doi: 10.1371/journal.pone.0152441 (PMC4822815; doi:10.1371/journal.pone.0152441)
Supplement: S2 Table — (DOCX) [file pone.0152441.s005.docx]

**S2 Table. Association between serum 25(OH)D and risk of cancer, Lappe cohort (N=1169)**

|  | **Unadjusted Hazard Ratio (95% CI)** | **p-value** | **Adjusted**^a^ **Hazard Ratio (95% CI)** | **p-value** |
| --- | --- | --- | --- | --- |
| **Serum 25(OH)D (ng/ml)** |  |  |  |  |
| <20 | Reference |  | Reference |  |
| 20-39 | 0.63 (0.28,1.42) | 0.27 | 0.59 (0.25,1.36) | 0.22 |
| ≥40 | 0.44 (0.14,1.39) | 0.16 | 0.46 (0.14,1.53) | 0.21 |

^a^Adjusted for age, BMI, smoking status, and calcium supplement intake.
